# Extramedullary Plasmacytoma of the Maxillary Sinuses in a Patient With Multiple Myeloma

**DOI:** 10.1002/oto2.144

**Published:** 2024-06-11

**Authors:** Caleb Boehler, Hasan Ozgur, Christopher Le, Samuel Rogers

**Affiliations:** ^1^ Department of Medical Imaging University of Arizona Tucson Arizona USA; ^2^ Department of Otolaryngology–Head and Neck Surgery Tucson USA

**Keywords:** extramedullary plasmacytoma, maxillary sinus, multiple myeloma

In patients with multiple myeloma (MM), extramedullary disease manifests as a plasmacytoma representing neoplasms of monoclonal plasma cell tumors either arising from bone or extraosseous soft tissue. This should not be confused with solitary extramedullary plasmacytoma, which arises in the absence of other features of MM. Extramedullary manifestations of MM are found in 13% to 15% of cases with 6% of cases being diagnosed on follow‐up and are associated with worse outcomes.^1^ Extramedullary plasmacytoma can be found in the soft tissues surrounding the skeleton, as well as other sites including lymph nodes, solid organs, lungs, pleura, and central nervous system (CNS). While it is common for solitary extramedullary plasmacytoma to involve the head and neck region, and cases of solitary extramedullary plasmacytoma have been reported in the maxillary sinuses, extramedullary manifestations in the head and neck are relatively rare in those patients with MM.^2^, ^3^ We present a unique case of sinonasal extramedullary plasmacytoma presenting in a patient with MM and symptoms of nasal obstruction, initially undiagnosed secondary to the absence of fluorodeoxyglucose (FDG) uptake on routine positron emission tomography/computed tomography (PET/CT) restaging.

## Case Report

**Figure 1:**
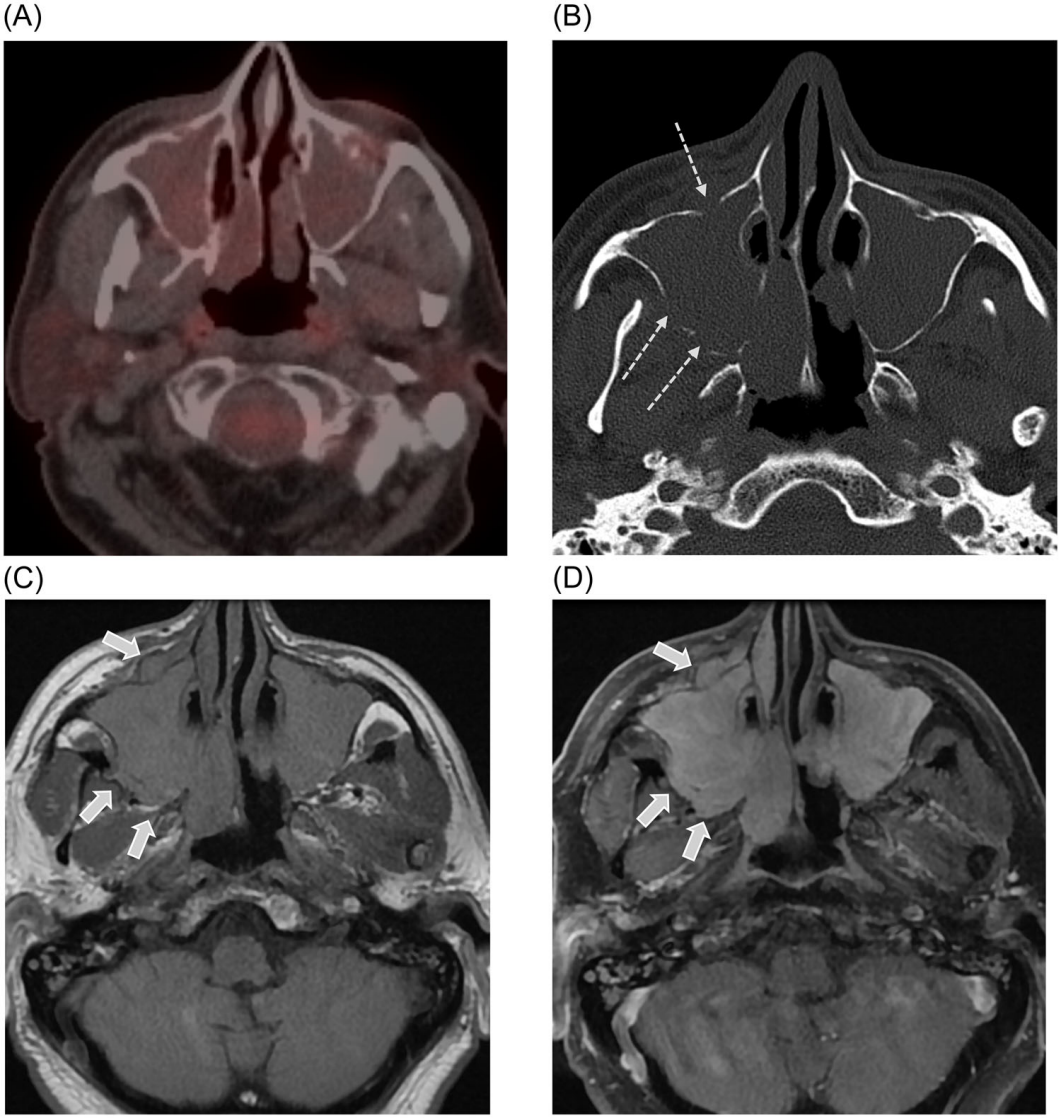
Positron emission tomography/computed tomography (PET/CT), sinus CT, and magnetic resonance imaging (MRI) images in a 74‐year‐old male with a history of multiple myeloma on maintenance chemotherapy for multiple myeloma, presenting with nasal congestion. (A) PET/CT shows diffusely opacified bilateral maxillary sinuses, without significant fluorodeoxyglucose uptake, interpreted as benign. (B) Follow‐up sinus CT 1‐month later shows progressive osseous dehiscence (thin dashed arrows), worrisome for a malignant process. (C, D) Follow‐up MRI precontrast and postcontrast T1 weighted images shows diffuse homogeneous enhancement.

A 74‐year‐old male with a past medical history of MM diagnosed in 2015, on maintenance chemotherapy, underwent follow‐up FDG PET/CT for MM staging in August of 2022, which demonstrated nonavid bilateral maxillary sinus disease, interpreted as benign. Subsequently, in September of 2022 he sought medical care for symptoms of bilateral nasal congestion, right greater than left, with obstructed nasal flow, facial pain and pressure, discolored drainage, and foul taste and odor. He had been experiencing these ailments since January 2022, at which time he was diagnosed with COVID‐19. His symptoms initially improved with nightly use of Afrin and Xylitol. However, he developed refractory symptoms and saw an ENT provider with no improvement after a course of antibiotics and prednisone. He was referred to ENT at our institution in September. Endoscopic endonasal examination revealed bilateral nasal masses with pulsatile and vascular character. Sinus CT and contrast‐enhanced brain and orbit magnetic resonance imaging (MRI) were obtained for further evaluation and revealed progressive osseous erosion and dehiscence of the maxillary sinuses with bilateral enhancing masses demonstrating trans‐osseous extension. Due to the physical exam and aggressive appearance on MRI and CT, the main differential diagnoses were a sinonasal primary malignancy and plasmacytoma, despite the lack of FDG avidity on the prior PET/CT. Biopsy of the lesion was deferred over concerns for bleeding given the vascular nature of the tumor. Surgery was expedited. The patient subsequently underwent endoscopic and open surgical resection, which confirmed the presence of bilateral maxillary sinus and anterior ethmoid sinus soft tissue with pterygopalatine fossa and skull base involvement. The frozen section was consistent with plasmacytoma with final pathology confirming kappa‐restricting plasma cell neoplasm. (Figure 1).

## Discussion

This is a rare and unique case of nonavid FGE PET/CT bilateral maxillary sinus extramedullary plasmacytoma in a patient with MM. Due to physical exam and imaging findings, the differential diagnosis included plasmacytoma or potentially new sinonasal primary malignancy. We demonstrate here that clinicians should be aware that suspicious lesions which are not FDG‐avid may still represent extramedullary involvement of MM.

The development of extramedullary disease in patients with MM is relatively uncommon and confers a poorer prognosis. Furthermore, extramedullary involvement of the paranasal sinuses in patients with prior MM is rarer still. Diagnosis of sinonasal extramedullary disease can present challenges both clinically and on imaging. Specifically, CT imaging features are nonspecific, typically limited to a soft tissue mass with associated bony destruction. MR is recommended to better evaluate the extent of disease. The typical MR findings of EMP include mild T1 signal hyperintensity, iso‐ hyperintensity on T2 weighted images, with homogeneous post‐contrast enhancement. PET/CT is recommended by the international Myeloma Working Group as a highly sensitive and specific modality to assess for active disease, however it may have false negative results in 10% of cases.^4^

The optimum treatment of sinonasal extramedullary plasmacytoma has not been sufficiently validated, largely due to its rarity. A variety of therapies have been attempted, both in combination and isolated, including surgery, chemotherapy, and radiotherapy. Of these options, surgery alone appears to offer the best clinical outcomes, with higher 1‐, 5‐, and 10‐year survival rates, slightly outperforming surgery with adjuvant radiotherapy. Both options significantly outperformed chemotherapy alone or combination radiotherapy and chemotherapy.^5^

## Author Contributions

**Caleb Boehler**, DO, primary author editing; **Hasan Ozgur**, MD, attending, manuscript editing; **Christopher Le**, MD, FACS, involvement in the surgical case, manuscript editing; **Samuel Rogers**, MD, attending, manuscript editing.

## Disclosures

### Competing interests

None.

### Funding source

None.
